# Influence of ZnF_2_ and WO_3_ on Radiation Attenuation Features of Oxyfluoride Tellurite WO_3_-ZnF_2_-TeO_2_ Glasses Using Phy-X/PSD Software

**DOI:** 10.3390/ma15062285

**Published:** 2022-03-19

**Authors:** Aljawhara H. Almuqrin, M. I. Sayyed

**Affiliations:** 1 Department of Physics, College of Science, Princess Nourah bint Abdulrahman University, P.O. Box 84428, Riyadh 11671, Saudi Arabia; ahalmoqren@pnu.edu.sa; 2 Department of Physics, Faculty of Science, Isra University, Amman 11622, Jordan; 3 Department of Nuclear Medicine Research, Institute for Research and Medical Consultations (IRMC), Imam Abdulrahman bin Faisal University (IAU), P.O. Box 1982, Dammam 31441, Saudi Arabia

**Keywords:** oxyfluoride tellurite glasses, gamma radiation, attenuation

## Abstract

The radiation shielding features of the ternary oxyfluoride tellurite glasses were studied by calculating different shielding factors. The effect of the TeO_2_, WO_3,_ and ZnF_2_ on the tested glass system’s attenuating performance was predicted from the examination. The mass attenuation coefficient (µ/ρ) values for the oxyfluoride tellurite glasses depend highly on the concentration of WO_3,_ as well as ZnF_2_. All the present ZnFWTe1-ZnFWTe5 samples have higher µ/ρ values than that of the pure TeO_2_ glass at all energies. For the samples with a fixed content of WO_3_, the replacement of TeO_2_ by ZnF_2_ increases the µ/ρ, while for the glasses with a fixed content of TeO_2_, the replacement of WO_3_ by ZnF_2_ results in a decline in the µ/ρ values. The results revealed that ZnFWTe4 has the lowest linear attenuation coefficient (µ) among the oxyfluoride tellurite glasses, whereby it has a slightly higher value than pure TeO_2_ glass. The maximum effective atomic number (Z_eff_) is found at 0.284 MeV and varied between 31.75 and 34.30 for the tested glasses; it equaled to 30.29 for the pure TeO_2_ glass. The half-value layer (HVL) of the glasses showed a gradual decline with increasing density. The pure TeO_2_ was revealed to have thicker HVL than the selected oxyfluoride tellurite glasses. A 1.901-cm thickness of the sample, ZnFWTe1, is required to decrease the intensity of a photon with an energy of 0.284 MeV to one-tenth of its original, whereas 1.936, 1.956, 2.212, and 2.079 cm are required for glasses ZnFWTe2, ZnFWTe3, ZnFWTe4, and ZnFWTe5, respectively.

## 1. Introduction

In the current century, radiation protection is becoming mandatory for non-ionizing radiation, such as infrared and microwave, as well as for ionizing radiation in several technological applications. Hence, it is necessary to perform empirical research that determines the shielding properties of several materials. In the construction of nuclear and industrial facilities where radioisotopes are planned for utilization, apart from the architectonic design and the normally evaluated mechanical, thermal, and physical features of the materials used in the construction, their photon-shielding characteristics are also important [1,2,3,4]. Radiation-shielding factors for the constructing facilities where gamma rays are used should be accurately determined and reported. Concrete is one of the most traditional materials utilized to shield from ionizing radiation, especially in medical applications, where X-rays are used in diagnosing patients [5]. Moreover, several kinds of rocks have been developed as radiation protection materials at different gamma energies from several keV to 10 MeV [6]. Moreover, glasses have been developed recently and utilized as promising shielding materials [7,8,9,10].

The comparatively cheap cost of preparing the glasses, their ability to be fabricated into any shape according to the applications, as well as their diverse methods for preparation and notably good photon attenuation coefficients make them attractive materials for shielding aims [11,12,13,14]. The gamma rays’ shielding tendency of glasses is directly related to their density, hence glasses prepared with heavy metal oxides, such as PbO, WO_3_, Sb_2_O_3_, andBi_2_O_3_, can appropriately be used as gamma ray shields [15,16,17,18]. Moreover, different works have demonstrated that the thicknesses of the glass sample can be reduced by using certain types of heavy metal oxides with an appropriate composition. TeO_2_-based glass systems are a subject of interest for investigators, materials engineers, and glasses developers, due to their interesting physical and chemical characteristics such as a large transparency window, high linear and non-linear refractive indexes, low phonon frequency, and good photons’ attenuation ability [19]. Oxyfluoride glass systems, including those based on TeO_2_, are also important objects.

Incorporation of metal fluorides to the tellurite systems may improve the physical properties for the resulting glass systems. The estimation of gamma photons’ attenuation factors for the oxyfluoride glass systems is very useful for the development of novel shielding glasses. There are several techniques to estimate the photon attenuation factors for any shields, such as: (a) Experimental methods using the transmission geometry technique or any other appropriate setup; (b) the numerical method, including different Monte Carlo simulation codes; and (c) the theoretical approach, using some common software [20,21,22]. In this research work, the radiation-shielding features of the WO_3_-ZnF_2_-TeO_2_ glasses were studied by calculating different shielding factors using the Phy-X/PSD software. Moreover, the role of the TeO_2_, WO_3_, and ZnF_2_ on the attenuating performance of the tested samples was predicted.

## 2. Materials and Method

It is well known that the gamma-ray attenuating characteristics of any medium depends on its composition and its density. For multi-component glass samples (such as the tested ternary oxyfluoride tellurite glasses), the mass attenuation coefficient (*µ/ρ*) can be found using Equation (1):

(1) $${\left(\mu /\rho \right)}_{glass}=\underset{i}{\sum }w_{i}{\left(\mu /\rho \right)}_{i}\;$$

In the above formula: *w_i_* is the respective weight fraction of the *i*th component (in this study, *w_i_* denotes the weight fraction of Zn, O, F, Te, and W). The linear attenuation coefficient (*µ*) is another factor that indicates the fraction of attenuated gamma rays when they pass into a medium. It is measured in a unit of cm^−1^ or mm^−1^. It is a density-dependent parameter and also an energy-dependent parameter. The aforementioned parameter is important, as it helps in determining other shielding parameters, such as the half value layer (HVL). It represents the width of the shield, where 50% of the incoming radiation has been attenuated [23]. Similar to µ, HVL is photon energy-dependent. The following formula is used for the evaluation of the HVL of any attenuator:

(2) $$HVL=\frac{0.693}{\mu }\;\;\;\;$$

Moreover, the mean free path (MFP) is another factor used by the shielding glasses developers to estimate the distance that the photons travel into the glass sample between collisions [24]. For practical utilization, especially where space is restricted, a glass sample with a small HVL, as well as MFP, is preferable. This can be obtained using dense samples that contain heavy metal oxides, such as WO_3_ and TeO_2_. For the tested WO_3_-ZnF_2_-TeO_2_ glasses, the following formula can be used for the evaluation of MFP:

(3) $$MFP=\frac{1}{\mu }\;\;\;\;\;$$

Moreover, for the selected oxyfluoride tellurite glasses, the researcher determined the effective atomic number (Z_eff_). This describes the interaction of radiation with composite materials. High Z_eff_ values for the sample imply the good shielding ability of the sample. The Z_eff_ for the tested oxyfluoride tellurite glasses was evaluated using Phy-X/PSD computer program [25]. This is a recently launched friendly online software that can estimate several radiation-shielding factors (such as Z_eff_) in the continuous energy region or chosen energy values (such as the energies emitted from the common radioisotopes). Any researcher can find this software online at https://phy-x.net/PSD (accessed on 1 January 2021).

In short, the method for the calculations of any shielding parameters for a certain sample using this software can be summarized as follows: (i) definition of the sample: the user must define the composition of the sample with its density. This is available in the software by using either the weight fraction or mole fraction. The weight (mole) fractions must equal to 1 (100). In this program, the user can define an unlimited number of samples at the same time by using the symbol (+) in the main screen of the program. The second step (ii) selection of the investigated energies: in this step, the user can define the energies at a wide energy range, such as 15 keV–15 MeV and 1 keV–100 GeV, or at some energies emitted from common radioisotopes, such as 0.356 MeV, 0.662 MeV, and 1.173 MeV. The third step (iii) selection of the parameters to be evaluated: in this software, the user can determine several parameters related to the radiation shielding at the same time. After these three steps, users can save the results in a Microsoft Office Excel file for further discussion and analysis. More details about the application language, main screen interface, and flow chart for this software are available in Ref. [25].

The compositions of the investigated glasses are listed in Table 1 [19,26]. Moreover, featured in the same table, is the density of the pure TeO_2_ glass. The selected samples were labeled as ‘ZnFWTe1’, ‘ZnFWTe2’, ‘ZnFWTe3’, ‘ZnFWTe4’, and ‘ZnFWTe5’, respectively, for convenience.

## 3. Results and Discussion

**Figure 1:**
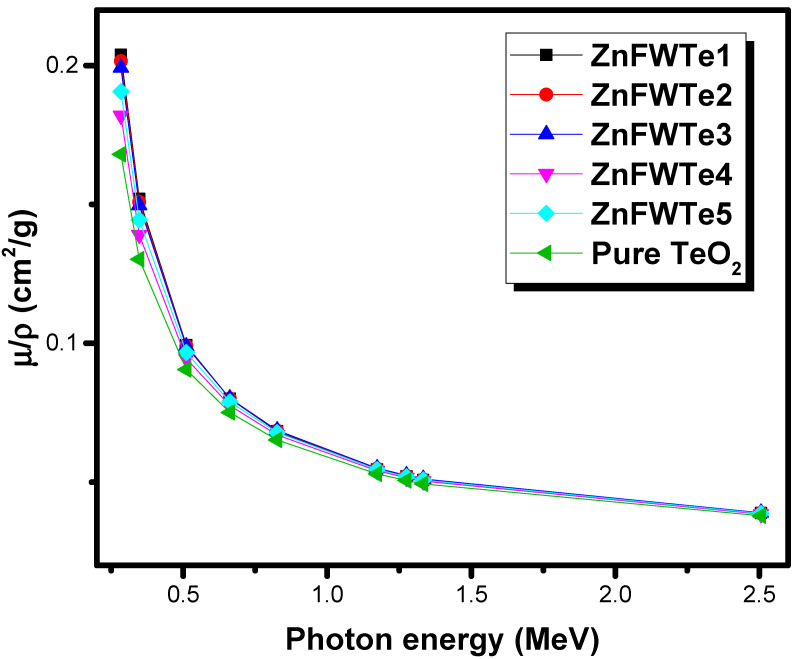
The mass attenuation coefficient for the oxyfluoride tellurite glasses.

The µ/ρ values have been evaluated by applying the Phy-X software at eight energies between 0.284–2.506 MeV (see Figure 1). Moreover, the µ/ρ for the pure TeO_2_ glass at the investigated energies is plotted in the same figure. As expected, the µ/ρ for the chosen glasses depends highly on the concentration of WO_3,_ as well as ZnF_2_. All the ZnFWTe1–ZnFWTe5 samples have higher µ/ρ values than that of the pure TeO_2_ glass. At 0.284 MeV, the µ/ρ values are 0.204, 0.202, 0.199, 0.182, and 0.191 cm^2^/g for ZnFWTe1, ZnFWTe2, ZnFWTe3, ZnFWTe4, and ZnFWTe5, respectively. Meanwhile, the µ/ρ for the pure TeO_2_ glass at this energy is 0.168 cm^2^/g.

In addition, at 0.826 MeV, the µ/ρ for the ZnFWTe1-ZnFWTe5 glasses are 0.0683, 0.0685, 0.0686, 0.0669, and 0.0678 cm^2^/g; whereas, for the pure TeO_2_ glass, it is 0.0652 cm^2^/g. From Figure 1, it can also be noted that for the samples with a fixed content of WO_3_ (namely ZnFWTe1, ZnFWTe2, and ZnFWTe3, which contain 20 mol% of WO_3_), the replacement of TeO_2_ by ZnF_2_ increases the µ/ρ, which is correct at all energies except at 0.284 MeV. Hence, the researcher found that (µ/ρ) _ZnFWTe3_ > (µ/ρ) _ZnFWTe2_ > (µ/ρ) _ZnFWTe1._ On the other hand, for the glasses with a fixed content of TeO_2_ (namely ZnFWTe1 and ZnFWTe4, which contain 70 mol% of TeO_2_), the replacement of WO_3_ by ZnF_2_ led to a decrease in the µ/ρ values. For instance, at 1.173 MeV, the µ/ρ for ZnFWTe1 and ZnFWTe4 are 0.0546 and 0.0541 cm^2^/g. For the samples with a fixed concentration of ZnF_2_ (i.e., ZnFWTe1 and ZnFWTe4), the decrease in WO_3_ content leads to a reduction in the µ/ρ values. The change in the µ/ρ may be ascribed to the µ/ρ of the constituent component, wherein the general WO_3_ has higher µ/ρ values than that of TeO_2_ and ZnF_2_, while ZnF_2_ has the smallest µ/ρ.

Notwithstanding, the µ can be utilized to deduce the fractions of photons that attenuated when passing through the glasses [27]. Consequently, it can assist in understanding the photon-attenuating trend for the oxyfluoride tellurite glasses. It has been proposed that the µ improves with increasing the density of the absorber [28]. The µ values for the oxyfluoride tellurite glasses are higher than that for the pure TeO_2_ glass. For example, the µ values for the selected oxyfluoride tellurite glasses at 0.284 MeV are 1.211, 1.190, 1.177, 1.041, and 1.108 cm^−1^ for ZnFWTe1, ZnFWTe2, ZnFWTe3, ZnFWTe4, and ZnFWTe5 glasses, respectively. The µ for the pure TeO_2_ glasses at this energy is 0.808 cm^−1^.

**Figure 2:**
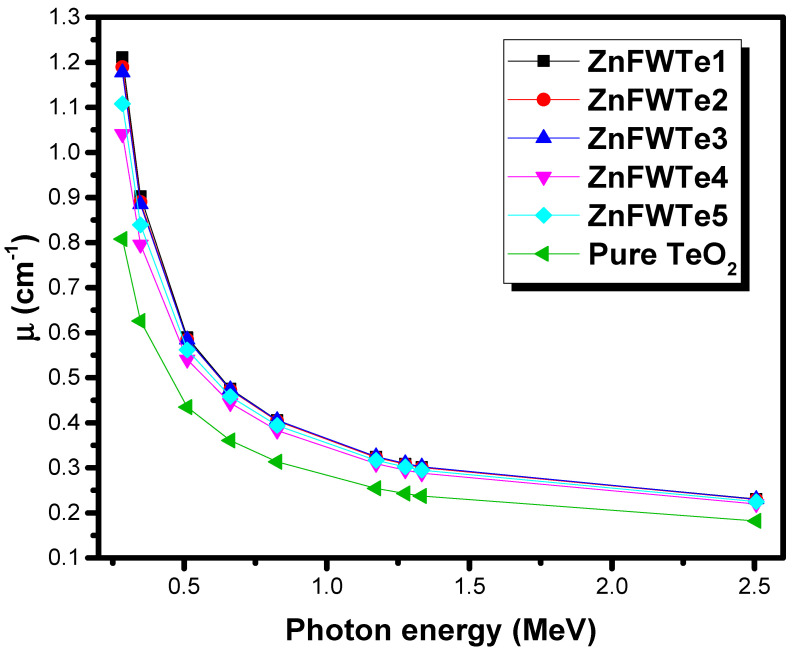
The linear attenuation coefficient for the selected oxyfluoride tellurite glasses.

For E = 0.826 MeV, the µ values are 0.406, 0.404, 0.406, 0.383, and 0.394 cm^−1^, and 0.313 cm^−1^ for the pure TeO_2_ sample. For all tested glasses (oxyfluoride tellurite glasses and pure TeO_2_ glass), the maximum attenuation behavior is found at 0.284 MeV, due to the photoelectric effect. Correspondingly, due to this process, one can see that the µ reduces quickly between 0.284 MeV and 0.662 MeV. For instance, for ZnFWTe1 and ZnFWTe2 samples, the µ varied between 1.211–0.476 cm^−1^ and 1.190–0.473 cm^−1^, respectively. Between 1.173–1.33 MeV, the Compton scattering becomes important, and, due to this process, the µ shows almost constant values with the increasing energy. For ZnFWTe1, the µ values are 0.324 cm^−1^ at 1.173 MeV, 0.309 cm^−1^ at 1.275 MeV, and 0.301 cm^−1^ at 1.33 MeV. From Figure 2, it could be observed that ZnFWTe1 has the highest µ at all considered energies. The glass ZnFWTe4 has the lowest µ among the oxyfluoride tellurite glasses. As expected, µ is a function of the density of the samples, whereby the µ shows a gradual increase with increasing the density. This result is similar to the findings reported for different glasses [29,30].

**Figure 3:**
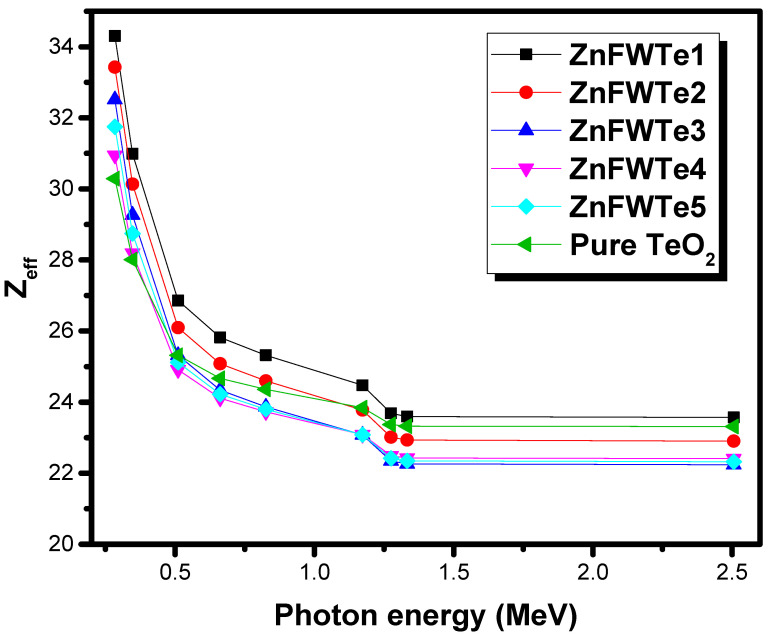
The effective atomic number for the selected oxyfluoride tellurite samples.

Figure 3 shows the Z_eff_ for the oxyfluoride tellurite glasses between 0.284–2.506 MeV. Moreover, in this figure, the researcher included the Z_eff_ for the pure TeO_2_ glass. As noted in the previous curves, the maximum Z_eff_ is found at 0.284 MeV and equals to 34.30, 33.42, 32.51, 30.94, 31.75, and 30.29 for ZnFWTe1, ZnFWTe2, ZnFWTe3, ZnFWTe4, ZnFWTe5, and pure TeO_2_ glass, respectively. The high Z_eff_ at this energy is related to the photoelectric effect (PE), and the possibility of PE depends upon Z^4–5^ and has a considerable inverse relation with the energy. Largely, one can see that Z_eff_ decreases with energy except at 2.506 MeV. The Z_eff_ behavior with the energy can be divided as follows: (i) a quick decreasing in Z_eff_ between 0.284–0.662 MeV, (ii) a very slight decrease in Z_eff_ between 0.826–1.33 MeV and (iii) a slight increase in Z_eff_, which occurs at the last energy only. For instance, for ZnFWTe1, the Z_eff_ in the previous three regions varied between 34.30–25.82, 25.32–23.57, and 23.57–24.47. Increasing the Z_eff_ in the last energy is related to pair production, as mentioned by Al-Hadeethi et al. [31].

**Figure 4:**
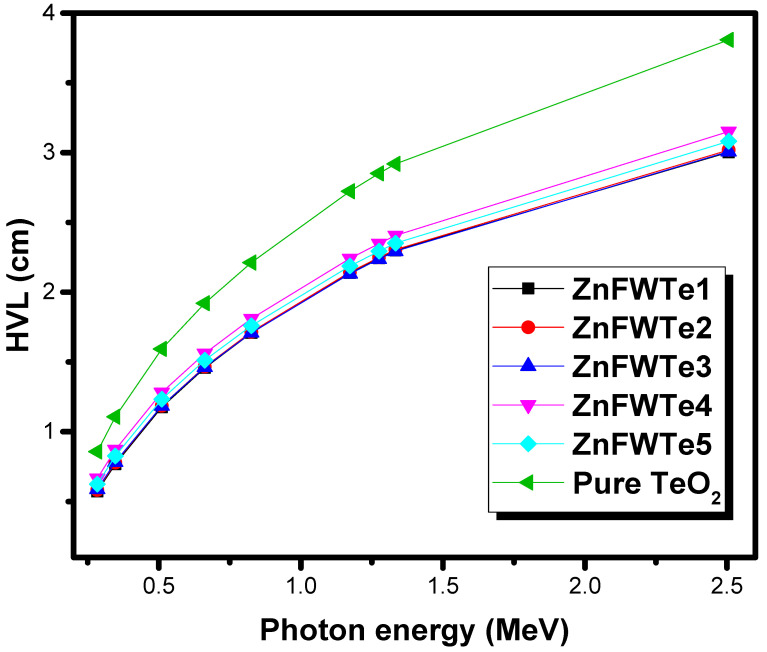
The half value layer for the selected oxyfluoride tellurite glasses.

Figure 4 presents the HVL for the chosen ZnFWTe1-ZnFWTe5 glasses and pure TeO_2_ glass at the eight energies under study. The increase in the density of the sample modifies the radiation-shielding properties of the samples. As expected, from Figure 4, the HVL depends strongly on the density of the samples, where ZnFWTe1 with the highest density possesses the least HVL and vice versa. The HVL of the glasses shows a gradual decrease with increasing the density. Following these grounds, it was found that the pure TeO_2_ has thicker HVL than the selected oxyfluoride tellurite glasses. At 0.284 MeV, the following values for the HVL were reported: 0.572, 0.583, 0.589, 0.666, and 0.626 cm for ZnFWTe1, ZnFWTe2, ZnFWTe3, ZnFWTe4, and ZnFWTe5. On the other hand, the HVL for pure TeO_2_ at this energy was established to be 0.858 cm. Moreover, the HVL shows a gradual increase with increasing energy. For ZnFWTe2, the HVL changes from 0.583 to 3.018 cm between these energies. Meanwhile, for pure TeO_2_ glass, the HVL changes from 0.858 to 3.808 cm. These results imply the weakening in the photons’ attenuating ability of the samples along with the increase in the energy of the photon. These results are consistent with those found earlier for different types of glasses [32,33].

**Figure 5:**
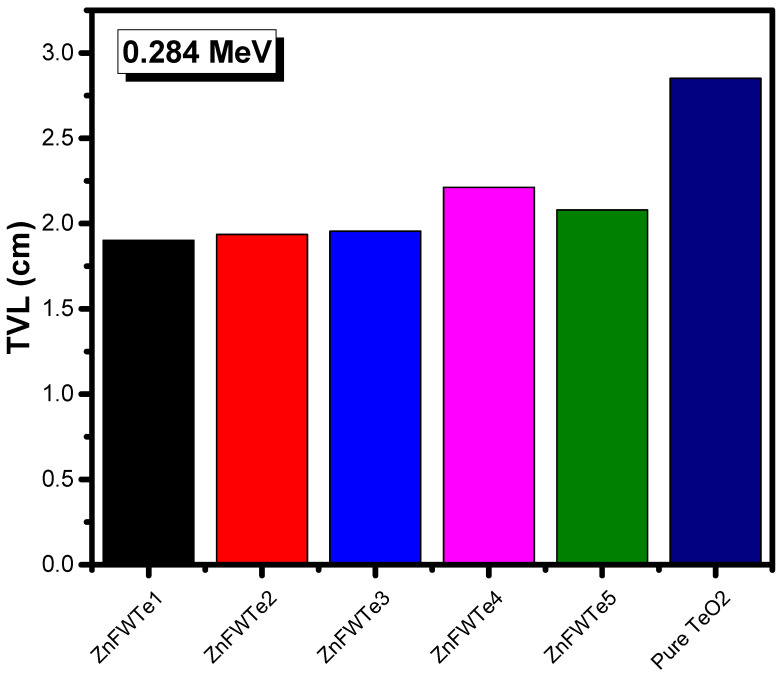
The tenth value layer for the selected oxyfluoride tellurite glasses at 0.284 MeV.

**Figure 6:**
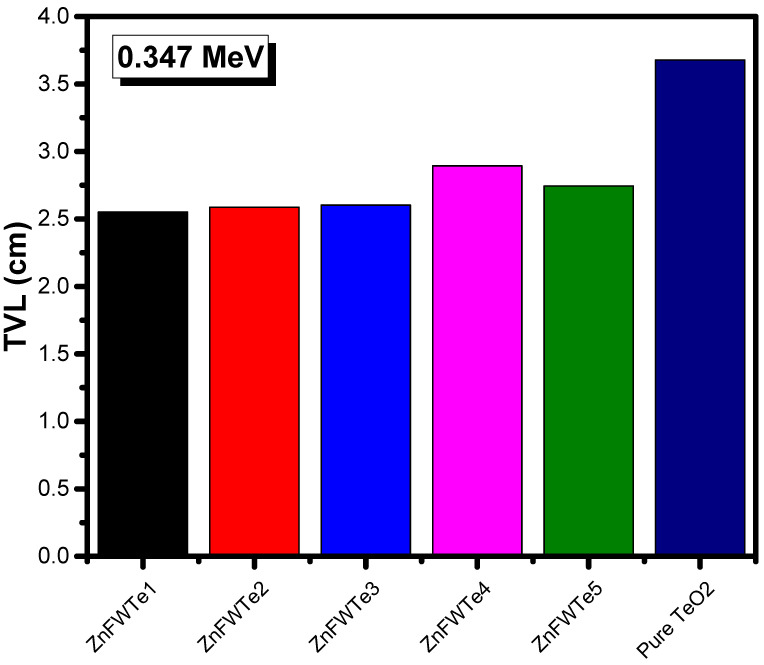
The tenth value layer for the selected oxyfluoride tellurite glasses at 0.347 MeV.

Moreover, we evaluated the TVL for the oxyfluoride tellurite glasses. The researcher plotted the TVL results for ZnFWTe1-ZnFWTe5 glasses and pure TeO_2_ glass at 0.284 and 0.347 MeV (as an example) in Figure 5 and Figure 6, respectively. It can be noticed from both figures that as the density of the samples increased, the TVL reduced and thus the photons’ shielding capability were enhanced. All the ZnFWTe1-ZnFWTe5 samples have a higher density than that of pure TeO_2._ Consequently, they have lower TVL than pure TeO_2_. This is correct at both energies, as represented in Figure 5 and Figure 6 and also at the other energies (not shown in this work).

At 0.284 MeV, for instance, one requires a 1.901-cm thickness of the sample ZnFWTe1 to decrease its intensity to one-tenth of its original (i.e., TVL), whereas 1.936, 1.956, 2.212, and 2.079 cm are required for glasses ZnFWTe2, ZnFWTe3, ZnFWTe4, and ZnFWTe5, respectively. It will take about a 2.851-cm thickness of pure TeO_2_ glass to achieve the same purpose. At 0.347 MeV, approximately a 2.550-cm thickness of ZnFWTe1 is required to reduce the incoming photons’ level to one-tenth of the original level, while 2.587, 2.603, 2.894, and 2.744 cm are required for the samples ZnFWTe2, ZnFWTe3, ZnFWTe4, and ZnFWTe5, respectively. For the pure TeO_2_ glass, a 3.678-cm thickness of this sample is required to achieve this aim. From these results, the researcher found that the TVL increases with the increasing energy.

**Figure 7:**
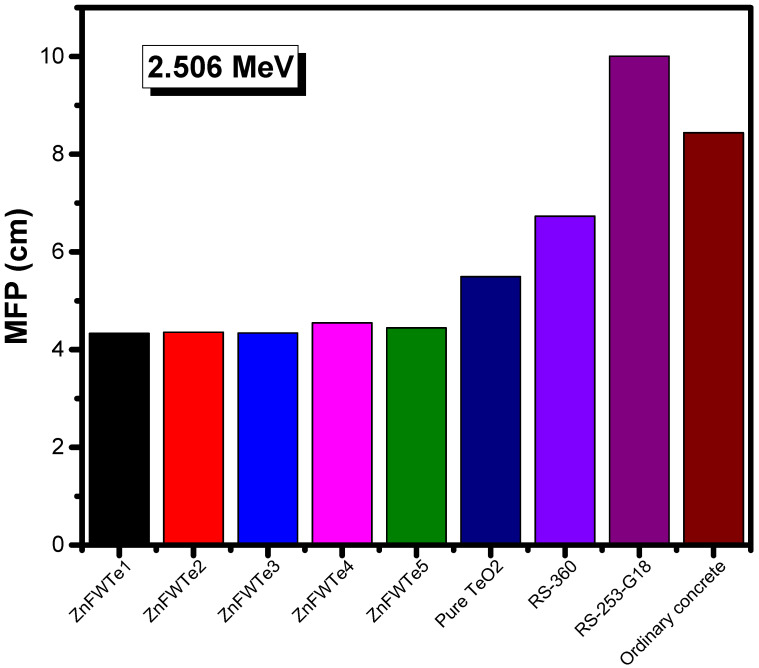
The mean free path for the selected oxyfluoride tellurite glasses at 2.506 MeV in comparison with other shielding materials.

Figure 7 illustrates the comparison between the MFP for the tested ZnFWTe1-ZnFWTe5 at 2.506 MeV with some common materials used for the radiation-shielding applications [34]. In general, it is expected that the MFP and µ should show an opposite trend to each other. This is true for the tested oxyfluoride tellurite glasses, as illustrated in Figure 7, whereby pure TeO_2_ has a higher MFP than the present ZnFWTe1-ZnFWTe5 glasses. Moreover, the current glasses have lower MFP than ordinary concrete and the RS-360 and RS-253-G18 glasses.

The HVL for the selected TeO_2_ glasses containing ZnF_2_ and WO_3_ at 0.347 MeV varied between 0.768 and 0.826 cm; this is lower than the HVL for 90.4 TeO_2_–9.6 ZnO-4NiO glass, which is equal to 0.967 cm [35]. Moreover, the HVL for the present glasses are slightly lower than those of the ZnO-MoO_3_-TeO_2_ glasses [36]. Rammah et al. [37] found that the HVL TeO_2_-Li_2_O-ZnO glasses at 0.347 MeV varied between 1.039 and 1.12 cm and this is higher than the HVL for our present glasses. Ersundu et al. [38] studied the WO_3_-MoO_3_-TeO_2_ glasses and for this glass system, the HVL is varied, at between 0.875 and 0.994 cm (at 0.347 MeV); thus, it shows a higher HVL than our present glasses. This confirms the possibility of developing the current glasses for radiation protection aims in the tested energy zone.

## 4. Conclusions

We reported the radiation shielding features of the ternary oxyfluoride tellurite glasses, WO_3_-ZnF_2_-TeO_2_, using Phy-X/PSD software. A discussion and prediction were provided concerning the effect of the TeO_2_, WO_3_, and ZnF_2_ on the attenuating performance of the tested glass system. The µ/ρ for the WO_3_-ZnF_2_-TeO_2_ glasses highly depends on the concentration of WO_3,_ as well as on ZnF_2_. All ZnFWTe1-ZnFWTe5 glasses have higher µ/ρ values than that of the pure TeO_2_ glass at the selected energies. The replacement of TeO_2_ by ZnF_2_ in the samples with a fixed content of WO_3_ increases the µ/ρ, as well as for the glasses with a fixed TeO_2_ content. In contrast, the replacement of WO_3_ by ZnF_2_ led to a decline in the µ/ρ values. ZnFWTe4 has the lowest µ among the oxyfluoride tellurite glasses; however, it has a higher value of µ than pure TeO_2_ glass. The maximum Z_eff_ is found at 0.284 MeV and varied between 31.75 and 34.30 for the tested glasses. The HVL of the current glasses shows a gradual decrease with the increasing density. The study found that the pure TeO_2_ has a thicker HVL than the selected samples. We found that a 1.901-cm thickness of ZnFWTe1 is needed to reduce the intensity of the photons with the energy of 0.284 MeV to one-tenth of its original, whereas 1.936, 1.956, 2.212, and 2.079 cm are required for the glasses ZnFWTe2, ZnFWTe3, ZnFWTe4, and ZnFWTe5, respectively.

## Figures and Tables

**Table 1:** Composition of the chosen ZnF_2_–WO_3_–TeO_2_ glass system.

Sample Code	ZnF_2_	WO_3_	TeO_2_	Density (g/cm^3^)
ZnFWTe1	10	20	70	5.94
ZnFWTe2	20	20	60	5.90
ZnFWTe3	30	20	50	5.91
ZnFWTe4	20	10	70	5.72
ZnFWTe5	25	15	60	5.81
Pure TeO_2_ glass				4.806

## Data Availability

Not applicable.
